# Preparing Health Professionals for Environmental Health and Climate Change: A Challenge for Europe †

**DOI:** 10.3390/healthcare14020208

**Published:** 2026-01-14

**Authors:** Guglielmo M. Trovato, Camille A. Huser, Lynn Wilson, Giovanni S. Leonardi

**Affiliations:** 1 The European Medical Association, 1160 Brussels, Belgium; giovannileonardi333@gmail.com; 2 School of Medicine, Dentistry and Nursing, The University of Glasgow, Glasgow G12 8QQ, UK; camille.huser@glasgow.ac.uk (C.A.H.); lynn.wilson@glasgow.ac.uk (L.W.)

**Keywords:** environmental health, climate change, medical and health-professional education, sustainable healthcare, curriculum development, Europe

## Abstract

Even though environmental health and climate change are rapidly intensifying the severity of determinants of disease and inequity, training for health professionals in these areas remains fragmented across Europe. To address this gap, the European Medical Association (EMA), in collaboration with the European Network on Climate and Health Education (ENCHE), the International Network on Public Health and Environment Tracking (INPHET) and University College London, convened a one-day hybrid roundtable in London on 17 September 2025, focused on “Preparing Health Professionals for Environmental Health and Climate Change: A Challenge for Europe”. The programme combined keynote presentations on global and European policy, health economics and curriculum design with three disease-focused roundtables (respiratory, cardiovascular and neurological conditions), each examining the following topics: (A) climate and environment as preventable causes of disease; (B) healthcare as a source of environmental harm; and (C) capacity building through education and training. Contributors highlighted how environmental epidemiology, community-based prevention programmes and sustainable clinical practice can be integrated into teaching, illustrating models from respiratory, cardiovascular, surgical and neurological care. EU-level speakers outlined the policy framework (European Green Deal, Zero Pollution Action Plan and forthcoming global health programme) and tools through which professional and scientific societies can both inform and benefit from European action on environment and health. Discussions converged on persistent obstacles, including patchy national commitments to decarbonising healthcare, isolated innovations that are not scaled and curricula that do not yet embed sustainability in examinable clinical competencies. The conference concluded with proposals to develop an operational education package on environmental and climate health; map and harmonise core competencies across undergraduate, postgraduate and Continuing -professional-development pathways; and establish a permanent EMA-led working group to co-produce a broader position paper with professional and scientific societies. This conference report summarises the main messages and is intended as a bridge between practice-based experience and a formal EMA position on environmental-health training in Europe.

## 1. Introduction

A global landscape of educational and professional training in environmental health (EH) has emerged, covering the health impacts of climate and other environmental change, as well as pollution. Efforts to implement the considerable evidence available build on the Alma Ata Declaration that health is a fundamental human right and that primary healthcare is key to delivering practical, scientifically sound and socially acceptable methods and activities, including environmental health interventions. There is a need to achieve convergence of multiple diverse approaches to education and training in climate and environmental health, encompassing approaches that strengthen factual knowledge and competencies to support diagnosis, treatment and prevention at the individual and societal level. These varied methodologies contribute to the development and practice of health professionals, making them more aware of preventable causes of illness, and help public health systems recognise ecological and environmental determinants of health. However, there is still a lack of consistent implementation of training programmes on climate and environmental health for clinical professionals, public health practitioners and other disciplines, and an absence of standardised curricula for undergraduates.

To address these gaps, a conference on this theme was organised by the European Medical Association (EMA) [1,2] in collaboration with the European Network on Climate and Health Education (ENCHE) [3], the International Network on Public Health and Environment Tracking [4] and the Institute for Global Health at University College London (UCL) [5], and was held at the latter’s premises in London on 17 September 2025. The conference provided a forum for making progress in this area within a broad European region and the UK. Participants included healthcare practitioners, public health operators, academic clinicians and health professionals responsible for undergraduate education, postgraduate training and continuing professional development (CPD). This report summarises the main messages of the meeting as an input to the EMA Working Group active on this theme.

The programme for the event is attached in Supplementary Materials. The event included three round tables focused on disease models for respiratory, cardiovascular and neurological conditions. Each area considered three themes: (A) climate and environment as preventable causes of disease; (B) healthcare as a source of harm to climate and environment; and (C) how to roll out effective capacity building to address (A) and (B) through training programmes.

### 1.1. Conference Sections

#### 1.1.1. Overview of Presentations and Discussions

Professor Vincenzo Costigliola, MD, President of the European Medical Association, opening the meeting, underlined the EMA’s longstanding commitment to international collaboration and invited all present to contribute actively to building a shared European medical culture on environmental and climate health.

Giovanni Leonardi, chair of the conference, on behalf of the International Network of Public Health and Environment Tracking (INPHET), reminded participants of two objectives for the day: first, to identify and compare existing curricula for climate and environmental health across different settings, looking for areas of convergence; and second, to translate this into practical tools such as shared learning units, practice units and mutual recognition mechanisms that could support mobility and the scaling up of education for health professionals. He situated this in ongoing WHO work to define environmental-health professional profiles, noting that environmental health is currently “hidden” within the 12 Essential Public Health Functions under health protection, which is still dominated by infectious disease budgets, and that WHO is seeking to guide countries on the dedicated environmental-health roles and competencies needed across sectors.

Sir Andy Haines’ keynote presentation “Environmental Health Capacity: Integrating Pollution and Climate Agendas” showed how actions that cut fossil fuel use create powerful synergies by simultaneously reducing greenhouse gas emissions and air pollutants, thereby preventing premature deaths while mitigating climate risks. He emphasised that decision-makers’ capacity for ecological sustainability improves when health, pollution and climate impacts are quantified together using integrated modelling, health-impact assessments and real-world policy evaluations. By combining high-quality environmental and health datasets and applying evaluations designed with multiple policy inputs and positive, health-framed communication, policymakers can adopt scalable, health-promoting and zero-carbon strategies aligned with windows of political opportunity. In the discussion, participants raised questions about how much Anthropocene and systems thinking medical students needed to know, the value of joint teaching with other disciplines, and how to motivate students who have grown up seeing planetary crisis as “normal” by giving them concrete, public health-oriented ways to act as professionals and citizens.

Presenting the European Union perspective, Joachim D’Eugenio (European Commission, DG Environment) described the wide policy framework linking the environment and health—from the European Green Deal and the Zero Pollution Action Plan to new air-quality standards, stricter wastewater rules for pharmaceuticals and cosmetics, and the “one substance, one assessment” system that integrates data from multiple EU agencies. These frameworks create clear entry points for professional and scientific societies to influence EU institutions by feeding clinical and epidemiological expertise into public consultations, highlighting how environmental determinants should be integrated into cancer, mental health and cardiovascular prevention strategies, and helping to define research priorities on issues such as multiple chemical exposures, microplastics and water resilience. Societies can, in turn, benefit from collaboration with EU institutions by using Commission and EEA tools (for example, the environment–health atlas and climate-risk assessments) in education and guideline development, drawing on robust EU data on inequalities and vulnerabilities, and partnering with EU-funded research or monitoring initiatives that strengthen curricula and clinical practice on the environment–health nexus.

Two presentations from UCL economists, Francesco Salustri and Ana Correa Ossa, examined health economics aspects of climate and environmental change. They emphasised that climate and environmental degradation are creating escalating economic pressures on health systems by increasing demand for care, emergency responses and management of pollution- and climate-related diseases. Prevention and preparedness, including environmental-health competencies, were presented as economically efficient investments that reduce hospitalizations, productivity losses and chronic disease costs. The speakers showed how greening healthcare—through energy efficiency, reduced waste and low-carbon clinical practices—can produce measurable savings, and how health economics can quantify the costs of inaction and assess the cost-effectiveness of interventions such as clean-air zones, green infrastructure and renewable energy in hospitals. They also stressed the importance of accounting for healthcare’s own carbon footprint in economic evaluations, enabling more accurate assessments of technologies and policies and supporting fiscally sustainable strategies for climate-related health challenges.

#### 1.1.2. Roundtable on Respiratory Disease

Elisa Puzzolo (University of Liverpool) showed how air pollution is now a leading global cause of disease and death, affecting both the global North and South, with especially heavy burdens in South Asia and sub-Saharan Africa. She stressed that risk factors such as biomass cooking, open waste burning and poorly designed housing make air quality a built-environment and infrastructure problem as much as a clinical one. Drawing on the CLEAN-Air (Africa) programme, she presented Kenya’s national Community Household Air-Pollution Prevention programme (CHAPP), which embeds air pollution prevention into the standard training curriculum for community health workers, enabling them to deliver behaviour-change, clean-energy and safety messages directly to households, including hard-to-reach nomadic communities. This model of integrated clinical and community-based practice—where doctors take an environmental exposure history, advise patients on reducing exposure, and work in partnership with community and social-care workers—offers lessons for Europe on how to combine sustainable clinical practice with upstream action on housing, energy and urban planning. Puzzolo underlined that air pollution control is both a health-equity and climate intervention, and that existing WHO training toolkits could be rapidly adapted into national curricula for health professionals.

Speaking as President of the European Public Health Alliance (EPHA), Paolo Lauriola underlined the central role of civil society advocacy in achieving sustainable healthcare for respiratory and other diseases. He described the EPHA as a broad alliance of NGOs, patient groups, researchers and professional organisations that use evidence-based advocacy to link primary-care realities—such as the everyday burden of asthma, COPD, infections and antimicrobial resistance—to wider European policies on climate, pollution, housing, transport and equity. By promoting education, training and capacity-building for family doctors and paediatricians, and by pushing for better environmental and health data systems, the EPHA works to ensure that clinical practice is supported by upstream policies that reduce exposure and vulnerability. Lauriola argued that this kind of organised civic pressure is essential for the social transformation required to move towards a climate-resilient society, where health and equity are systematically integrated into decisions on energy, urban planning and economic development.

#### 1.1.3. Roundtable on Cardiovascular Disease

Ariana Zeka (UK Health Security Agency) used cardiovascular disease models to show how environmental epidemiology and public health are essential to designing sustainable healthcare. She explained how population-based tools—such as global burden-of-disease analyses, exposome frameworks and long-term cohort studies—reveal multi-causal pathways linking air pollution, toxic metals, temperature extremes and other environmental risks to cardiovascular outcomes, and how these risks are unevenly distributed across populations. Public health’s focus on disease time-course, upstream exposures and organised efforts of society complement clinical work at the individual level: epidemiology identifies key environmental precursors, vulnerable groups and effective policy and system-level interventions that can both reduce disease burden and address inequalities. Integrating this epidemiological evidence into medical education and practice—through better exposure histories, investigative skills and advocacy for risk reduction—was presented as a cornerstone of sustainable healthcare, enabling health professionals to act not only on the sick individual but also on wider population risks that drive demand for care.

Gabriella Captur (UCL/Royal Free Hospital) used her experience as a cardiologist and cardiac MRI specialist to show how sustainability is reshaping core clinical skills in cardiovascular care. She described how cardiology now increasingly includes taking an environmental exposure history (air quality, heat, housing) and adapting treatment—for example, counselling heart-failure patients on heat risks or reconsidering high-impact investigations—so that clinicians learn to balance patient benefit with environmental footprint. Captur illustrated how this feeds directly into education: teaching trainees the “5Rs” hierarchy (refuse, reduce, reuse, repurpose, recycle) for high-impact procedures such as MRI, promoting contrast-free imaging approaches that avoid gadolinium pollution, and arguing that competencies in stewardship and system literacy (understanding hospital energy use, procurement and waste streams) must become part of routine training, CPD and revalidation for cardiovascular professionals.

#### 1.1.4. Roundtable on Neurological Disease

Gabriele De Luca (University of Oxford) described how undergraduate neurology teaching had been reshaped to link clinical brain health with planetary health. Starting from the high global burden and inequities of neurological disease, and the barriers created by stigma and “neurophobia”, he designed a teaching approach that makes neurology accessible while opening discussion of environmental and climate determinants of brain disease. Using expert patients with Parkinson’s disease, multiple sclerosis and peripheral neuropathies, students rotate through short bedside encounters to train in simple but powerful examination tools, gain confidence in diagnosis and discuss environmental risk factors such as infection, heat, air pollution, pesticides and extreme weather. De Luca framed brain health as a life-course concept linked to diet, green space, active transport and reduced harmful exposures, complementing clinical teaching with leadership sessions and the “Green Brain Capital” concept to help students see their role in shaping healthier, more sustainable systems.

In a very focused perspective, Kenneth Barker (NHS Scotland) framed sustainable surgical practice around two linked priorities: cutting the carbon and pollution footprint of operating theatres and embedding environmental stewardship into everyday clinical decisions. He showed how anaesthetic-gas choices, energy-hungry theatre ventilation, oversized instrument sets and single-use plastics all drive avoidable emissions, waste and microplastic contamination that ultimately harm patients and populations. His methods for change start from grassroots clinician initiatives, such as phasing out desflurane and nitrous oxide, and scaling up into the national Green Theatre Programme, which has developed a broad set of actions including switching off theatre ventilation when not in use, streamlining instrument trays and replacing single-use items with reusables. These actions are embedded in NHS Scotland governance, with chief executives reporting on implementation. Barker stressed the importance of education through a “Once for Scotland” curriculum in which sustainability is woven through teaching for doctors, nurses and allied professionals, and students and trainees are actively involved in audits, data collection and quality-improvement projects.

In a wider perspective, San YuMay Tun (University of Oxford) outlined how efforts to build environmental and planetary health into health-professional curricula have been driven by regulatory, ethical and conceptual shifts, such as the UK General Medical Council’s new sustainability duty in Good Medical Practice, a research-based definition of sustainable healthcare that links high-quality care with protecting ecosystems, and the recognition of planetary health as a transdisciplinary, action-oriented movement that seeks to reduce the need for healthcare by tackling upstream drivers of disease. She described how these drivers led to the UK Education for Sustainable Healthcare curriculum framework—topic-based and context-adaptable—endorsed by the Medical Schools Council and used by the GMC to inform graduate outcomes, and to an international AMEE consensus statement positioning planetary health within mainstream professional education [6]. Tun highlighted concrete steps to embed these themes into routine teaching: convening a national Medical Schools Council Alliance with representatives from all UK medical schools, sustained faculty development, use of student-led tools such as the Planetary Health Report Card, and collaboration with regulators, royal colleges and clinical networks. She showed that embedding environmental health education requires working simultaneously “from above” (standards, position statements and curriculum documents) and “from below” (student projects, local audits, clinical skills lab reforms and deprescribing initiatives), so that planetary and environmental health become woven through the everyday curriculum for medical and other health-professional students rather than remaining an optional add-on.

#### 1.1.5. Summary of Discussions on Topics of Roundtables

Across the three roundtables, there was striking convergence on core propositions, alongside some nuanced differences in emphasis that enriched, rather than fractured, the debate. Speakers and discussants consistently agreed that environmental determinants—particularly air pollution, climate-amplified hazards and broader planetary disruption—are major, preventable drivers of respiratory, cardiovascular and neurological disease, and that healthcare itself contributes substantially to environmental harm through energy-intensive infrastructure, resource-heavy technologies, pharmaceuticals and pervasive single-use materials. There was also broad consensus that primary care, community health workers and specialist clinicians are under-used assets for prevention, surveillance and advocacy, and that significant, competency-based reforms in undergraduate, postgraduate and continuing professional education are required to embed environmental health, sustainable care and systems thinking as integral components of “good clinical practice” rather than optional add-ons. An overview of topics raised by speakers and discussants recurrently in the three clinical roundtables, grouped by thematic focus, is presented in Table 1.

Within this shared framing, participants articulated differing emphases regarding the pace and depth of curricular change, the balance between incremental integration and more transformative redesign, and the nature of engagement with commercial actors. Some contributors argued for radical, systemic reorientation of health professional education towards prevention, social and commercial determinants, and planetary health, while others foregrounded pragmatic, stepwise strategies using existing levers such as accreditation, examinations and quality-improvement requirements. Similarly, while there was agreement that commercial determinants of health—most notably fossil-fuel interests—must be confronted, views diverged on the scope for constructive collaboration with selected industry partners in education and innovation, with more cautious voices stressing structural conflicts of interest and others highlighting guarded opportunities for co-production under robust safeguards. Finally, discipline-specific perspectives (for example, on neurophobia and diagnostic overuse in neurology or on high-impact imaging and devices in cardiology) coloured how sustainable practice was operationalised, yet these were framed as complementary illustrations of a shared agenda to align clinical excellence with environmental stewardship across the health system.

#### 1.1.6. Overview of Presentations on Ways Forward

Drawing on experience across settings, professor Camille Huser (University of Glasgow) highlighted major obstacles to climate- and environment-competent health systems: patchy national commitments to decarbonising healthcare; innovations that remain isolated case studies rather than scaled policies; and curricula that stop at one-off lectures, leaving students at the level of “knowing about” sustainability rather than “doing” it in practice. Sustainable care is still rarely embedded in core clinical skills or assessments, so it is not yet treated as synonymous with excellent care. As ways forward, she argued for systematic integration of sustainable healthcare into practical skills, clinical reasoning and quality-improvement projects; making sustainability examinable, so it becomes part of core professionalism; stronger institutional commitments at university, national, European and global levels; and active use of networks such as ENCHE and WHO-aligned initiatives to share resources, harmonise competencies and ensure that education is explicitly included in upcoming climate–health action plans such as the Belem Health Action Plan at COP30.

On behalf of the EMA, Guglielmo Trovato explained that the Association’s main leverage over EU institutions lies in systematic, visible engagement with European Commission processes rather than informal lobbying. The EMA uses the Commission’s “Have Your Say” consultation mechanism to speak directly to Directorates-General, submitting comments on a wide range of initiatives and consistently inserting the need for environmental-health education, practical training and recognition of NGOs’ contributions. The EMA also organises visits to Commission buildings, provides suggestions rooted in clinical and public health practice, and amplifies these positions through its communication channels. In parallel, it develops position papers and cultivates relationships with members of the European Parliament so that climate- and environment-competent professional education becomes embedded in future EU legislation and action plans. These mechanisms will likewise support the dissemination of the forthcoming EMA Position Paper on environmental health training.

Based on topics where convergence between participants emerged at the conference, a set of possible actions at national and European/international levels could be identified, and these have been grouped by reference to topics within Theme C (educational and capacity-building) and are presented in Table 2.

## 2. Conclusions

In their concluding remarks, Prisco Piscitelli and Giovanni Leonardi suggested that the next steps should include developing an operational education package on environmental and climate health using existing tools and resources; presenting this in a coordinated way to rectors and deans of medical schools, starting with institutions already linked to the network; and exploring a permanent working group including the experts involved in the meeting to refine standardised curriculum proposals that can be adapted across countries and languages [7].

Professor Costigliola closed the meeting by thanking organisers, speakers and participants, emphasising that this was an excellent starting point rather than an endpoint, and expressing the hope that the gathering would lead to sustained collaboration and long-term joint work on this agenda, particularly in view of the implications for future generations.

## Figures and Tables

**Table 1:** Recurrent topics raised by speakers and discussants in three clinical roundtables, grouped by thematic focus.

	Theme (A): Climate/Environment as Preventable Cause of Disease	Theme (B): Healthcare as a Source of Harm to Climate and the Environment	Theme (C): How to Roll out Effective Capacity Building to Address (A) and (B) Through Training Programmes
**Respiratory roundtable**	Air pollution (ambient and household) as major, preventable driver of respiratory, cardiovascular and systemic disease, with strong equity gradients.	Healthcare’s carbon and pollution footprint (~5% of national emissions) and the need to view clinical care as part of the climate and pollution problem.	Integration of environmental health and climate content into medical curricula (standalone modules plus integration into physiology, pathology, clinical specialties).
	Climate amplified respiratory risks (heat, wildfires, ozone, aeroallergens) and disproportionate impact on children, elderly and disadvantaged groups.	Over investigation, over prescription and lack of deprescribing as drivers of avoidable environmental burden and patient harm.	Use of primary care- and community-based workers (e.g., CHW training modules such as CHAPP) as scalable models for prevention-focused environmental health training.
	Household air pollution (biomass/charcoal) and associated gender, child and socioeconomic inequities in exposure and health outcomes.		
**Cardiovascular roundtable**	Air pollution, temperature extremes and lead as key environmental risk factors for cardiovascular disease within a multi-causal exposome framework.	Resource-intensive cardiovascular care (surgery, intensive care, advanced imaging) as high impact sources of emissions and pollution requiring stewardship.	Embedding environmental history taking, prevention and sustainable clinical decision making into undergraduate and postgraduate cardiology training.
	Climate change as a modifier of cardiovascular risk through direct (heat/cold) and indirect (food, mental health, service disruption, social determinants) pathways.	Environmental impact of pharmaceuticals and contrast agents (e.g., gadolinium, other residues) via wastewater and aquatic contamination.	Competency-based, assessed education in sustainable healthcare (exposure pathways, stewardship, advocacy) as a requirement for professional standards and certification.
**Neurological roundtable**	Environmental determinants of brain disease (heat, air pollution, pesticides, extreme weather, infections) and their under-recognition, especially in climate-vulnerable regions.	Hospital infrastructure and theatre practice (HVAC, central vacuum, single-use plastics, anaesthetic gases) as major, avoidable sources of emissions and microplastic/chemical pollution.	Tackling “neurophobia” through patient-led, skills-based neurology teaching to reduce delays, misdiagnosis and associated environmental and patient harms.
	Brain-health behaviours (diet, physical activity, sleep, green space, social connection) as co-benefits for neurological and planetary health.	Diagnostic overuse and “diagnostic odysseys” in neurology (neurophobia-driven) contribute to unnecessary tests, travel and associated environmental impacts.	Framing “brain health” as a life course, prevention-oriented concept linked to planetary health to engage students and the public.
**All three roundtables**		Healthcare pollution (air, water, microplastics, chemicals) as a threat to current and future patients, with calls for extended producer responsibility and zero pollution policies.	Positioning sustainable, prevention-oriented care as core to “good clinical practice”, supported by regulators, royal colleges and medical associations.
			Building formal alliances and networks (e.g., ENCHE, INPHET, ESH alliances) to share curricula, resources and best practices on climate, environment and health.
			Interdisciplinary and inter-faculty education (medicine with architecture, engineering, planning, agriculture, economics) to address environmental determinants of health.

**Table 2:** Priority actions derived from convergent educational and capacity-building themes (Theme C) across three clinical roundtables.

Ref. to Table 1 (Theme/Roundtable/Topic)	Level	Action	Primary Actors/Sectors	Intended Outcome/Rationale
Respiratory/Integration of EH and climate content into curricula	National	Mandate inclusion of environmental health (EH) and climate–health learning outcomes in core undergraduate and postgraduate curricula (e.g., via national regulators, accreditation standards).	Ministries of Health and Education; national regulators; medical and nursing councils; universities	Ensure all new clinicians have baseline competencies in environmental determinants of health, prevention, and sustainable practice.
Respiratory/Integration of EH and climate content into curricula	European/international	Develop a shared European reference framework for EH and climate–health competencies (ECTS-compatible), usable by medical, nursing and public-health schools.	EU bodies (e.g., DG SANTE, DG EAC), European professional associations, ENCHE, INPHET	Facilitate mutual recognition of EH training, support curriculum convergence and cross-border mobility of professionals.
Respiratory/Community-based CHW training models (CHAPP)	National	Adapt the CHAPP-type community training model to national respiratory and EH priorities (e.g., air pollution, indoor exposures) and integrate into official CHW/primary-care curricula.	Ministries of Health; primary-care agencies; CHW training institutions; NGOs	Scale primary and secondary prevention by equipping frontline workers with practical skills on exposure reduction and health promotion.
Respiratory/Community-based CHW training models (CHAPP)	European/international	Include CHW-style prevention modules in European capacity-building programmes for neighbouring and partner countries (e.g., in EU external health programmes).	EU health and development programmes; WHO Regional Office; international NGOs	Transfer proven prevention models to low-resource settings, strengthening EH capacity and reducing environment-related respiratory burden.
Cardiovascular/Environmental history-taking and sustainable decision-making	National	Embed structured environmental history-taking (air, heat, work, housing, fuels) into core clinical skills teaching and OSCEs in cardiology and internal medicine.	Medical schools; specialist colleges; OSCE boards	Normalise environmental exposure assessment in routine cardiovascular care; enable earlier, targeted prevention and counselling.
Cardiovascular/Environmental history-taking and sustainable decision-making	European/international	Produce consensus teaching cases and OSCE scenarios on environmental determinants of cardiovascular disease for shared use across European training programmes.	European cardiology societies; ENCHE; specialty training bodies	Provide ready-to-use, harmonised educational material, reducing duplication and raising standards across countries.
Cardiovascular/Competency-based sustainable healthcare education	National	Require documented competencies in sustainable healthcare (including stewardship and advocacy) for specialty certification and revalidation in cardiology, general practice and internal medicine.	National royal colleges and specialty boards; regulators	Align professional incentives with sustainable practice; ensure sustainability is treated as a core component of “good practice”.
Cardiovascular/Competency-based sustainable healthcare education	European/international	Develop European-level competency statements for sustainable cardiovascular care and integrate them into European examination blueprints (e.g., ESC/EBAC curricula).	European cardiology and internal-medicine societies; accreditation councils	Promote consistent expectations of sustainable CVD care across member states and support cross-border recognition of expertise.
Neurological/Tackling “neurophobia” via patient-led skills teaching	National	Introduce structured, patient-tutor-led neurology skills sessions (e.g., “speed-dating” with expert patients) into all medical schools to reduce neurophobia and diagnostic delay.	Universities; neurology departments; patient organisations	Improve basic neurological competence among all graduates, reducing misdiagnosis, unnecessary investigations and associated environmental footprint.
Neurological/Tackling “neurophobia” via patient-led skills teaching	European/international	Create a shared toolkit (curriculum, videos, patient-tutor guidance) for neurology skills teaching linked to brain and planetary health, for translation and adaptation across countries.	European Academy of Neurology; ENCHE; patient federations	Enable rapid diffusion of effective neuro-education models and connect neurology training with environmental and planetary-health narratives.
Neurological/Brain-health concept linked to planetary health	National	Integrate “brain health across the life course” (including diet, physical activity, sleep, green space, social connection) into preventive medicine and neurology teaching.	Medical and public-health schools; neurology and psychiatry societies	Increase student and clinician engagement with prevention by linking personal brain performance, patient outcomes and climate-friendly behaviours.
Neurological/Brain-health concept linked to planetary health	European/international	Include brain-health and planetary-health co-benefits as a specific chapter in European public-health and neurology training recommendations.	European public-health and neurology associations; WHO Europe	Make brain-health promotion a visible component of European strategies on noncommunicable diseases and climate–health co-benefits.
All roundtables/Sustainable, prevention-oriented care as “good practice”	National	Embed sustainable, prevention-oriented care explicitly in national “Good Medical Practice” or equivalent codes and cascade into all health-profession codes.	National regulators; professional councils; ethics bodies	Reframe sustainability and prevention as ethical and professional duties, not optional extras.
All roundtables/Sustainable, prevention-oriented care as “good practice”	European/international	Develop a joint statement by European regulatory and professional bodies defining sustainability and prevention as core dimensions of professional competence.	European regulatory networks; medical and nursing associations; EMA	Provide a shared reference for member states, supporting aligned regulation and accreditation reforms.
All roundtables/Alliances and networks (e.g., ENCHE, INPHET, ESH alliances)	National	Support national EH and sustainable-healthcare networks (e.g., national ESH alliances, green-practice networks) with modest core funding and formal links to universities and health services.	Ministries of Health; national public-health institutes; professional bodies	Stabilise bottom-up initiatives, enhance coordination and allow systematic sharing of tools and curricula within countries.
All roundtables/Alliances and networks (e.g., ENCHE, INPHET, ESH alliances)	European/international	Strengthen and expand European networks (ENCHE, INPHET, professional alliances) as hubs for curriculum co-development, shared resources and multicountry pilots.	ENCHE; INPHET; European professional societies; EU agencies	Accelerate learning across countries, reduce duplication, and support coherent European responses on EH training.
All roundtables/Interdisciplinary and inter-faculty education	National	Establish joint teaching modules on environmental determinants of health involving medicine, public health, engineering, architecture, urban planning, agriculture and economics.	Universities; deans of multiple faculties; accreditation bodies	Build systems thinking and cross-sector collaboration skills needed to address complex climate and environmental health challenges.
All roundtables/Interdisciplinary and inter-faculty education	European/international	Promote inter-faculty, cross-country summer schools or intensive programmes on climate, environment and health, with ECTS recognition.	European universities; Erasmus+/similar schemes; professional associations	Create a pipeline of health professionals and partners trained to work across disciplines and borders on climate-health challenges.
All roundtables/Assessment and quality improvement as levers	National	Include sustainability-related stations and questions in national exams (written and clinical) and require at least one sustainability-oriented quality-improvement project in postgraduate training.	Exam boards; postgraduate training committees; royal colleges	Align learners’ effort with sustainability priorities; convert abstract concepts into practical, measurable change.
All roundtables/Assessment and quality improvement as levers	European/international	Develop banks of validated exam items and QI project templates on EH and sustainable care, shared among European training programmes.	European examination consortia; specialty boards; ENCHE	Support high-quality, comparable assessment of EH and sustainability competencies across countries and specialties.

## Data Availability

No new data were created or analyzed in this study.

## Supplementary Materials

The following supporting information can be downloaded at: https://www.mdpi.com/article/10.3390/healthcare14020208/s1, File S1.
