# Supplementary material for: Preparing Health Professionals for Environmental Health and Climate Change: A Challenge for Europe
Source: Healthcare (Basel). 2026 Jan 14;14(2):208. doi: 10.3390/healthcare14020208 (PMC12840772; doi:10.3390/healthcare14020208)
Supplement: Supplementary file 1 [file healthcare-14-00208-s001.zip › healthcare-4068811-supplementary.pdf]

European Medical Association

INPHET International Network on Public Health & Environment Tracking

EUROPEAN NETWORK ON CLIMATE & HEALTH EDUCATION

Preparing Medical Professionals on Environmental Health and Climate Change. A Challenge for Europe

London, 17th September 2025

The European Medical Association (EMA)
The European Network on Climate and Health Education (ENCHE)
International Network on Public Health and Environment Tracking (INPHET)
University College London (UCL Institute for Global Health)
Invite you to the conference at the
UCL Institute for Global Health, Room 411, 4th floor, The Mortimer Market Centre, Mortimer Market (off Capper Street), London WC1E 6JB.

FACULTY:

Chair: Giovanni Leonardi

President: Vincenzo Costigliola

Vice-President: Guglielmo Trovato.

Co-Chairs: Camille Huser, Francesco Salustri and Prisco Piscitelli

KeyNote Speakers:

Andy Haines, Joachim D'Eugenio, Elisa Puzzolo, Paolo Lauriola, Ariana Zeka, Gaby Captur, Kenneth Barker, Gabriele DeLuca, SanYuMay Tun, Ana Correa Ossa.

Microsoft Teams
Join the meeting now
Meeting ID: 386 844 448 312
Passcode: SL7Kf2Jg

OVERVIEW

The European Medical Association (EMA) in collaboration with European Network on Climate and Health Education (ENCHE), International Network on Public Health and Environment Tracking (INPHET), University College London (UCL Institute for Global Health)

Invites you to the conference on

Preparing Medical Professionals on Environmental Health and Climate Change

A Challenge for Europe

Date: 17th September 2025 in person and online

Venue: **UCL Institute for Gobal Health, Room 411, 4th floor, The Mortimer Market Centre, Mortimer Market (off Capper Street), London WC1E 6JB**.

OVERVIEW

A global landscape of educational and professional training in environmental health (EH) has emerged. It includes consideration of health impacts of climate and other environmental change, as well as pollution. Efforts to implement the considerable evidence available are ongoing in the light of the Alma Ata Declaration that health is a fundamental human right, and health care based on primary care is needed for implementing practical, scientifically sound and socially acceptable methods and activities including environmental health interventions. Training to achieve this encompasses a range of approaches to strengthening factual knowledge and competencies to support diagnosis and treatment at individual and societal level. These disparate offers in principle contribute to development and practice of health care professionals so they are more aware of preventable causes of illness, as well as to make public health systems more aware of ecological or environmental dimensions. In this field there is a lack of consistent global implementation of training programmes for clinical professionals, public health practitioners, and individuals across various disciplines, as well as of standardized curricula for undergraduates. The conference strives to provide a forum for making progress in this area within the European region and UK. Participants will include health care practitioners, public health operators, academic clinicians and health care workers responsible for undergraduate education and postgraduate training.

The event will include three round tables focused each on a disease model: respiratory, cardiovascular, and neurological. Each disease area will consider three themes: A). Climate/environment as preventable cause of disease, B). Healthcare as a cause of harm to climate/environment, C) How to roll out effective capacity building to address (A) and (B) by effective training programmes.

The overall aim is to address the question: “What opportunities can be identified for best practice arrangements identified to be included in (i) EH curricula in universities; (ii) EH training as part of professional practice in health and social care; (iii) EH training as part of professional practice in public health?”

Objectives:

- Converge toward the proposal of a specific curriculum on environmental determinants of health and on the effects of climate changes on human health for preparing/training health professionals (undergraduate and postgraduate) during their official course of studies;
- Compare different possible approaches and teaching methodologies such as creating dedicated “teaching units” (European Credit Transfer and Accumulation System (ECTS) on EH and/or

integrating EH issues into subjects already delivered to students (physiology, pathology, clinical medicine, clinical surgery, psychiatry etc).

Programme

9:30 – 10:00	Registration and coffee	
10.00-10.15	Venue presentation	Francesco Salustri (in person)
	Welcome to the day	Welcome by the President of EMA Vincenzo Costigliola (online)<br>Prisco Piscitelli (online)<br>and Camille Huser (online)
10.15-10.30	Key-note speech 1: "The contribution of economics to environmental health"	Francesco Salustri (in person)
10:30-10:45	Objectives of the day. "The need of new focused curricula in health, science and training. "	Giovanni Leonardi (Chair of the Conference, in person)
10:45-11:45	Keynote Lecture 1 "Environmental health capacity: need for integrating pollution and climate agendas" and discussion	Andy Haines (in person)
11:45-12.00	Comments and remark on the topic of the meeting.	Joachim D'Eugenio, Policy Adviser for Zero Pollution Strategy, DG Environment (ENV) (online)
12.00-13.00	LUNCH	
13.00-13:45	Round table 1 - Respiratory Disease	Elisa Puzzolo, University of Liverpool (Honorary Senior Research Fellow) (in person)<br>Paolo Lauriola, European Public Health Alliance (online)
13:45-14:30	Round table 2 – Cardiovascular Disease	Ariana Zeka, UK Health Security Agency (in person)<br>Gaby Captur, Cardiologist and Professor, The University College London (UCL) Institute of Cardiovascular Science & Royal Free London NHS Foundation Trust (in person)
14.30-15:15	Round table 3 – Neurological disease	Kenneth Barker, surgeon, lead of National Green Theatres Scotland (video)<br>SanYuMay Tun (in person)<br>Gabriele De Luca, Clinical Neurology and Experimental Neuropathology, Oxford (in person)
15:15-15:45	TEA/COFFEE	
15:45-16:00	Keynote speech 2, on "Overview on some of the current initiatives of	Guglielmo Trovato (in person)

European Medical Association

	the European Commission dealing with environment and health: contributions and suggestions of the European Medical Association”	
16:00-16:15	Keynote speech 3: “Health Economics and Decision Science proposing innovation in Environmental Health policies and curricula”	Ana Correa Ossa, Health Economist. University College London, Institute for Global Health (in person)
16.15-16.30	Keynote speech 4: Some issues related to environment care and current medical and surgical practice: educational proposals of focused curricula.	Camille Huser
16:30-16:45	Conclusions and proposals	Vincenzo Costigliola/Giovanni Leonardi/Prisco Piscitelli

FACULTY:

Chair: Giovanni Leonardi

Co-Chairs: Camille Huser, Francesco Salustri and Prisco Piscitelli

EMA President: Vincenzo Costigliola

EMA Vice-President: Guglielmo Trovato.

KeyNote Speakers:

Andy Haines

Joachim D’Eugenio

Elisa Puzzolo

Paolo Lauriola

Ariana Zeka

Gaby Captur

Kenneth Barker

Gabriele De Luca

Ana Correa Ossa

SanYuMay Tun

Lynn Wilson

If you are attending in person, please note the venue address: UCL Institute for Global Health, Room 411, 4th floor, The Mortimer Market Centre, Mortimer Market (off Capper Street), London WC1E 6JB.

The MS Teams details for online presenters and guests are:

____

Microsoft Teams Need help?

Join the meeting now

Meeting ID: 386 844 448 312

Passcode: SL7Kf2Jg

Dial in by phone

+44 20 3794 0272,,942422378# United Kingdom, City of London

0800 047 8623,,942422378# United Kingdom (Toll-free)

Find a local number

Phone conference ID: 942 422 378#
